# Opto-mechanical lab-on-fibre seismic sensors detected the Norcia earthquake

**DOI:** 10.1038/s41598-018-25082-8

**Published:** 2018-04-27

**Authors:** Marco Pisco, Francesco Antonio Bruno, Danilo Galluzzo, Lucia Nardone, Grzegorz Gruca, Niek Rijnveld, Francesca Bianco, Antonello Cutolo, Andrea Cusano

**Affiliations:** 1 Optoelectronic Division - Engineering Dept., University of Sannio, c.so Garibaldi 107, 82100 Benevento, Italy; 2 Istituto Nazionale di Geofisica e Vulcanologia, Osservatorio Vesuviano, via Diocleziano 328, 80124 Napoli, Italy; 3 OPTICS11 B.V., De Boelelaan 1081, 1081 HV Amsterdam, The Netherlands

## Abstract

We have designed and developed lab-on-fibre seismic sensors containing a micro-opto-mechanical cavity on the fibre tip. The mechanical cavity is designed as a double cantilever suspended on the fibre end facet and connected to a proof mass to tune its response. Ground acceleration leads to displacement of the cavity length, which in turn can be remotely detected using an interferometric interrogation technique. After the sensors characterization, an experimental validation was conducted at the Italian National Institute of Geophysics and Volcanology (INGV), which is responsible for seismic surveillance over the Italian country. The fabricated sensors have been continuously used for long periods to demonstrate their effectiveness as seismic accelerometer sensors. During the tests, fibre optic seismic accelerometers clearly detected the seismic sequence that culminated in the severe Mw6.5 Norcia earthquake that struck central Italy on October 30, 2016. The seismic data provided by the optical sensors were analysed by specialists at the INGV. The wave traces were compared with state-of-the-art traditional sensors typically incorporated into the INGV seismic networks. The comparison verifies the high fidelity of the optical sensors in seismic wave detection, indicating their suitability for a novel class of seismic sensors to be employed in practical scenarios.

## Introduction

Earthquakes occur frequently worldwide. Seismic events with various intensities are continuously revealed by seismic stations located in various parts of the world^1^. Most earthquakes cause vibrations that cannot be perceived by humans. However, the release of elastic energy associated with some earthquakes can generate seismic waves with sufficient force to damage buildings or trigger catastrophic collapses^2^.

During only the past ten years, several devastating earthquakes have occurred^3^. Although earthquakes occur worldwide, most are concentrated in high seismic risk zones located at tectonic plate boundaries. Italy, for example, lies at the contact point between tectonic plates, where intense tectonic movements currently induce seismic activity in geographical correspondence with the Apennine Mountains. As a consequence, strong earthquakes frequently strike central Italy (the Abruzzo region on April 2009, the Emilia-Romagna on May 20, 2012, and the Umbria, Lazio and Marche on Aug. 24, 2016) and have killed hundreds recently and injured many more. During one of these seismic sequences, a 6.5-magnitude quake struck Norcia on October 30, 2016. Fortunately, no one died as a result, but the medieval basilica of St. Benedict in Norcia, symbol of the historical heritage of this town, was severely damaged^4^.

Earthquakes are natural events that cannot be avoided, but an efficient monitoring system in the regions of high seismic risk is essential for the safety of millions of people living in these zones. A seismic network allows one to determine the frequency, energy, and physical phenomenology of the earthquakes that characterize a certain area. Additionally, a sensing network can provide useful data for seismologic studies, for risk assessment and for the potential development of future prediction strategies^5^. Furthermore, the growth of the seismic monitoring network, both in quantity and in the quality of the installed instruments, improves the network detection capability and the precision of earthquake epicentre identification. Just after a significant earthquake, indeed, temporary seismic networks are typically installed to integrate the existing permanent network in the epicentral area and to provide additional data for investigation into the physics of earthquakes^5,6^.

The implemented monitoring techniques are currently based primarily on seismometers, geophones, and accelerometers. These sensor typologies are complementary (and partially overlap) in terms of bandwidth and dynamic range, and when operated in conjunction, they supply detailed information on seismic events. Conventional technologies are generally well-suited, reliable and high-performance tools^7^, but severe drawbacks are associated with their practical use, especially when a high number of measurement points is required or the installation must be performed in open spaces. Indeed, conventional seismic sensors are heavy, bulky and challenging to transport; they are affected by electromagnetic interference, and their installation requires extensive wiring or a wireless network for data communication. The installation complexity grows substantially when a large number of sensors are deployed.

In this scenario, sensors based on optical fibre technology (OFT) can provide an alternative approach based on certain OF properties. OF sensors are typically light, small and immune to electromagnetic interference. The cabling is greatly simplified, and the fibre itself provides the necessary communication medium. The remote operability and multipoint sensing ability of OF sensors make them well suited for monitoring large areas and hostile environments, as the interrogation unit can be installed in a suitable and safe location^8,9^.

Although OF sensing networks have already been demonstrated for large structure monitoring and extensive multipoint measurement applications^10,11^, optical sensing technology for seismic vibration detection has still not been fully exploited. A few solutions have been proposed, involving the integration of fibre Bragg gratings (FBGs) with various mechanical structures, such as dual-cantilever beams^12,13^, an inverse pendulum^14^ and diaphragms^15–17^. These structures lie far from the realm of practical use because they cannot offer competitive performance in terms of sensitivity with respect to conventional seismic accelerometers. The performance limitations may be intrinsically related to the pursued approach in which a strain sensor is integrated with a bulk mechanical structure. The transduction efficiency (and thus the sensitivity) is detrimentally affected by the mechanical compliance of the structure (and the sensor itself).

An alternative approach to achieving the sensitivity required to detect small seismic movements is the integration of micromechanical structures and OFs.

OFT has already demonstrated its capability to be a strongly beneficial platform for developing multifunctional and miniaturized sensing devices. Lab-on-fibre (LOF) technology has found several uses in the chemical and biological fields by taking advantage of the integration of multiple structures and materials in confined domains to promote enhanced light-matter interaction^18,19^. Similarly, the integration of cavity optomechanics^20^ with OFT can pave the way for innovative micromechanical LOF devices that can be readily applied in realistic scenarios.

From the technological perspective, in the past decade, important steps forward have included the fabrication or integration of micromechanical structures on the OF tip.

Kilic *et al*.^21^ presented a micromachined OF microphone based on a photonic-crystal diaphragm placed at short distance from the fibre tip. Subsequently, the same authors fabricated an optical hydrophone based on a compliant membrane on the OF tip for underwater applications^22^.

In 2006, Iannuzzi *et al*. demonstrated the fabrication of micro-cantilevers onto the fibre end facet using focused ion beam instrumentation^23–25^. Subsequently, the same group proposed the use of a borosilicate glass ferrule to fabricate a cantilever suspended on the OF tip using a ps-laser ablation tool^26,27^. In addition, a top-down approach via align-and-shine photolithography^28^ was proposed to fabricate low-mass gold fibre-top cantilevers. Following these technological improvements, a wide variety of sensors has been demonstrated^29–33^.

Here, we propose a novel LOF seismic sensor based on opto-mechanical micro-cavities on the OF tip. To achieve this configuration, a double-beam cantilever was designed to meet the specific performance requirements of seismic accelerometer sensors. The designed sensors were fabricated using ferrule-top technology, and the resulting functionality was preliminarily verified in the laboratory. The fabricated sensors were then validated during field tests conducted at the INGV, the Italian National Institute devoted to continuous seismic surveillance over the country. During the field trial, the LOF sensors detected the Norcia earthquake of October 30, 2016. The practical use of the sensor for earthquake detection was successfully demonstrated by comparing the seismic trace recorded by the OF sensors with commercial seismic accelerometers.

## Methods

### Fibre optic seismic sensors: concept and modelling

**Figure 1:**
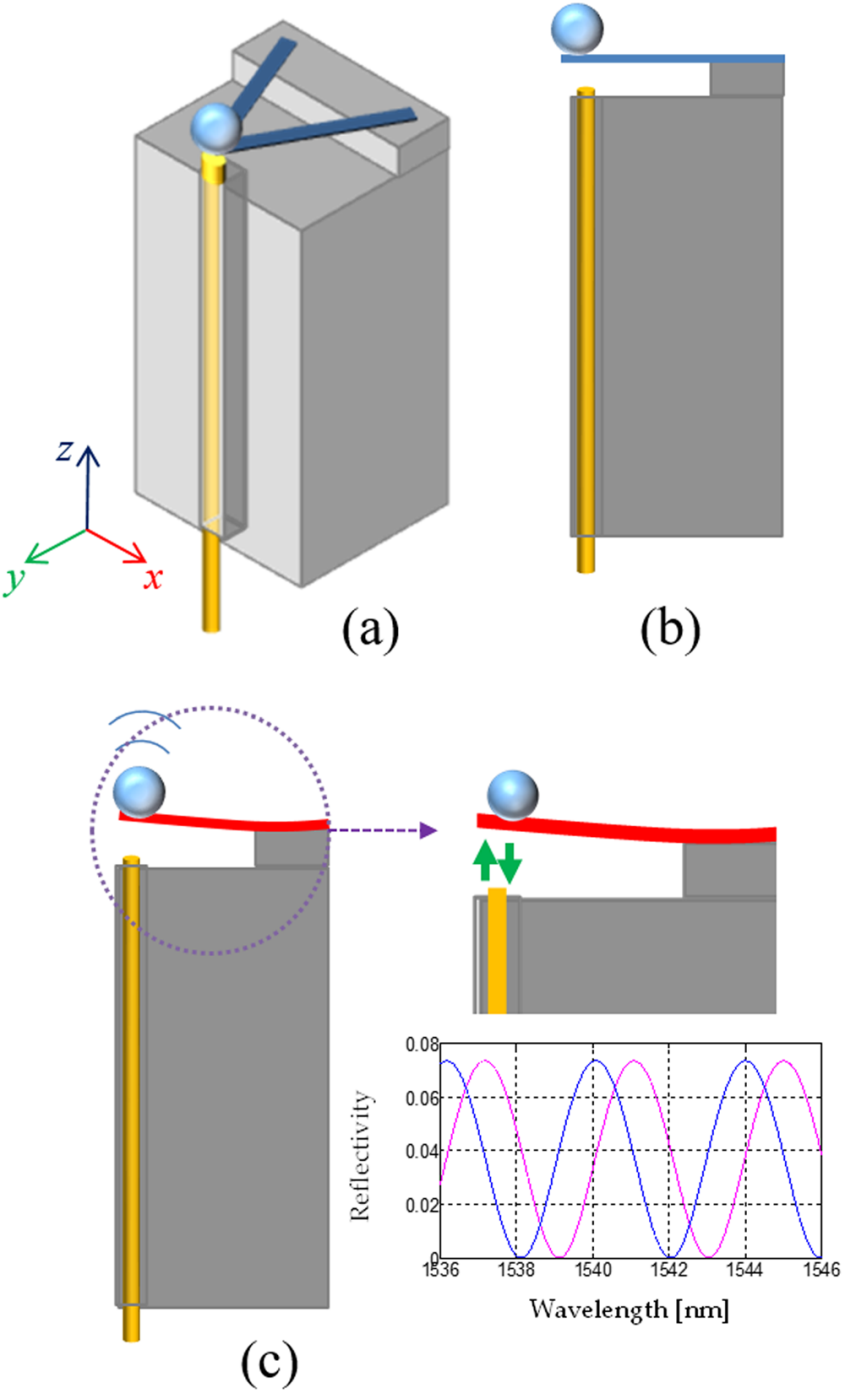
(**a**) Schematic of the OF seismic sensor; (**b**) Lateral view of the OF seismic sensor, (**c**) Schematic picture of the cantilever deflection due to a ground vibration. Inset: The Fabry-Pérot air cavity changes for effect of the cantilever deflection and an interferometer phase shift occurs in the reflectance spectrum.

The proposed LOF seismic sensor configuration relies on a V-shaped glass cantilever beam with an added mass at the free end; on the other side, both arms are clamped on the sensor body. As schematically shown in Fig. 1(a,b), the free end of the V-shaped cantilever is suspended above the OF end facet, while the OF is attached to the sensor body. The air cavity on the fibre tip behaves as an extrinsic Fabry-Pérot cavity for the light propagating in the OF. When a vibration occurs, the distance between the cantilever free end and the fibre end facet changes, and this change can be optically detected using interferometric interrogation techniques (see Fig. 1(c)).

The mechanical structure on the OF tip behaves essentially as an inertial seismic sensor that acts as a simple harmonic oscillator. A flexible cantilevered beam with an affixed mass on the end can be modelled as a mass-spring-damper system. The resulting system dynamic response can be expressed in the Fourier domain as follows (see the Supplementary Information):

1 $$H(\omega )=\frac{X(\omega )}{A(\omega )}=\frac{1/{\omega }_{R}^{2}}{1-{(\frac{\omega }{{\omega }_{R}})}^{2}+(2\varsigma \frac{\omega }{{\omega }_{R}})j}$$

where

*X(ω)* is the mass displacement with respect to the ground versus the harmonic frequency ω;

*A(ω)* is the ground acceleration to be detected;

${\omega }_{R}=\sqrt{\frac{k}{m}}$ is defined as the natural frequency and $\varsigma =\frac{c}{2\sqrt{k\cdot m}}$ is the damping ratio; and

*m* represents the mass, *k* the spring constant, and *c* the viscous damping coefficient.

**Figure 2:**
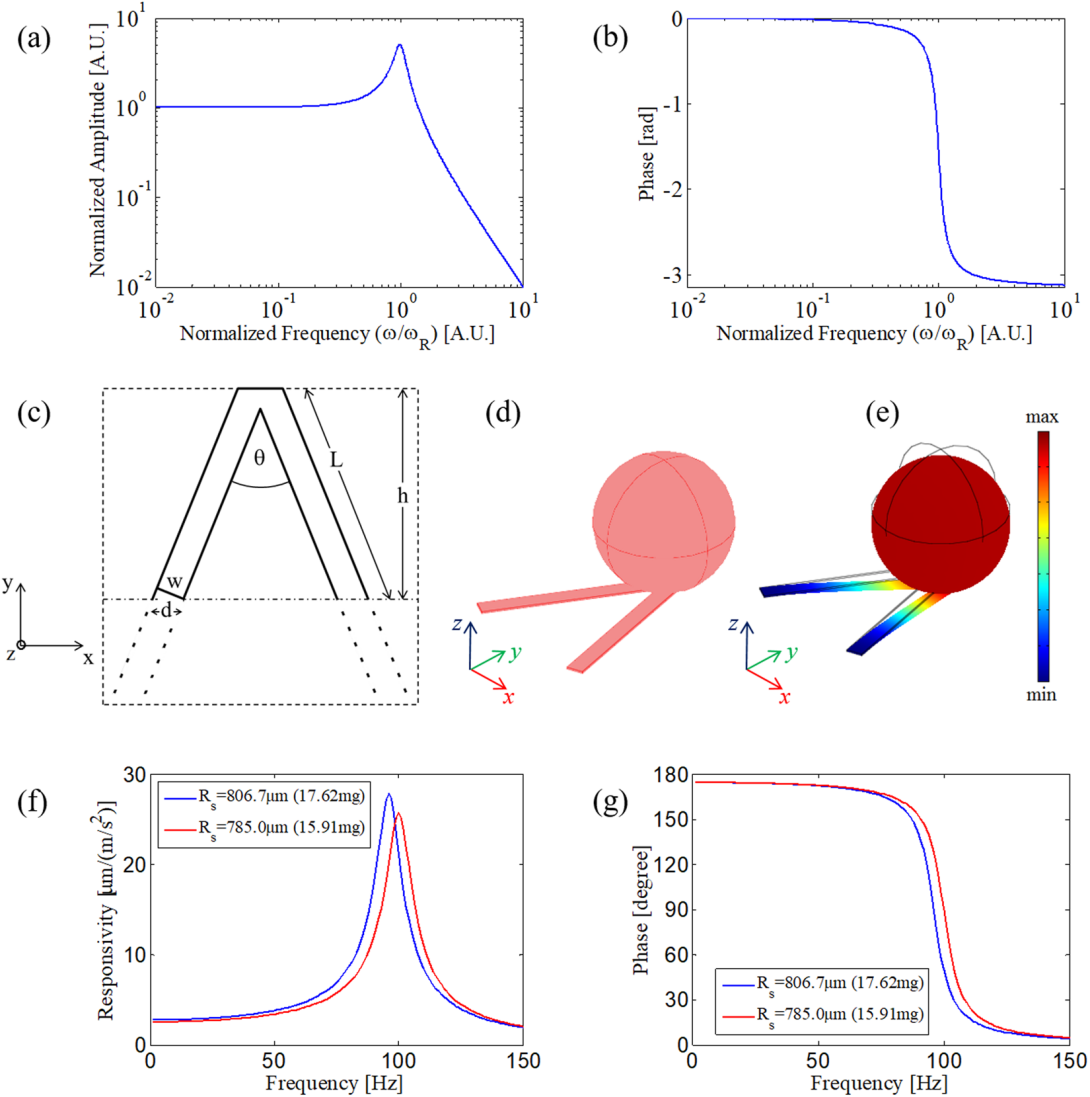
(**a**) normalized amplitude; and (**b**) phase of the system dynamic response versus the normalized harmonic frequency for a damping ratio of 0.1. (**c**) geometry of the cantilever beams; (**d**) computational domain of the V-shaped cantilever with added mass; (**e**) “deformed shape” representation of the total displacement when the structure is subject to a vertical acceleration at 5 Hz; (**f**) numerical responsivity in amplitude of the OF sensor; and (**g**) numerical responsivity of the phase of the OF sensor.

The system transient response is characterized by an exponential decay with a time constant $\frac{1}{\varsigma {\omega }_{R}}$. According to equation (1), at frequencies well below the natural frequency, *ω* ≪ *ω*_*R*_, the relative mass displacement is directly proportional to the acceleration. The proportionality factor can be defined as the in-band sensor responsivity ${S}_{0}=1/{\omega }_{R}^{2}$. Since the in-band responsivity *S*_0_ is inversely proportional to the natural frequency, a performance trade-off between the in-band responsivity and system bandwidth must be managed. Actually, in principle, even the damping ratio can affect the sensor bandwidth and the resonance magnitude. However, in a practical micromechanical system, the damping is low and depends primarily on the physical properties of the selected constitutive materials. To demonstrate the system behaviour, we display in Fig. 2(a,b) the harmonic response (*H*(*ω*)) normalized to the responsivity ${S}_{0}=1/{\omega }_{R}^{2}$, in amplitude and phase, versus the harmonic frequency *ω* normalized to the natural frequency *ω*_*R*_ for a damping ratio of 0.1.

### Fibre optic seismic sensor design

Seismic accelerometers are typically employed in a seismic network for “strong motion” detection, whereas relatively small ground movements are entrusted to seismometers.

We summarize in Table 1 the minimum requirements for the LOF accelerometer to be competitive with commercial accelerometer sensors used in seismic applications^34–37^. A resolution of at least $1\frac{\mu g}{\sqrt{Hz}}$ must exist^34^. A bandwidth ranging from 1 Hz up to 20 Hz can be considered sufficient to detect earthquakes because most of the earthquake spectral content is concentrated over this range^35^.

**Table 1:** Target performance for seismic accelerometers.

Parameter	Value
Minimum frequency	<1–2 Hz
Maximum frequency	>20–50 Hz
Resolution	$< 1\frac{\mu g}{\sqrt{Hz}}$
Settling time	~1–10 s

However, state-of-the-art seismic networks continuously require improved performance in terms of the sensor chain. Therefore, improved performance would be desirable in any case to extend the applicability of the seismic accelerometer sensors.

Of course, many other figures of merit affect the performance of the sensor, from the settling time (typically of the order of seconds or more^36,37^), the size, and the weight to more general properties, such as the electromagnetic immunity or the ability to multiplex the sensors.

We designed the micromechanical system to meet the basic requirements reported in Table 1 by taking advantage of the properties of OF sensing technology. To design the system, we assumed that the system is only slightly damped, and we selected the natural frequency to achieve a suitable trade-off between the bandwidth and sensor resolution, whereas we increased the damping ratio in the experimental stage to reduce the settling time ${T}_{S}\approx \frac{4}{\varsigma {\omega }_{R}}$ to acceptable values. By setting the natural frequency at 100 Hz, under the assumption of low damping ($\varsigma \approx 0$ in equation (1)), we obtain an approximately 10% flat bandwidth of 20 Hz and a 3 dB bandwidth of 40 Hz. The 100 Hz natural frequency, in turn, leads to an in-band responsivity of approximately $2.53\frac{\mu m}{(m/{s}^{2})}$. To obtain a resolution of $1\frac{\mu g}{\sqrt{Hz}}$, an interrogation readout is required that can detect displacements with a resolution down to $25\frac{pm}{\sqrt{Hz}}$. State-of-the-art interferometric readout units^38,39^ and even commercially available units^40,41^ can typically provide such resolution (or better). Finally, to achieve a settling time lower than 1 s, a damping ratio higher than 0.0064 is required.

To set the dimensions of the opto-mechanical cavity for exhibiting a natural frequency of 100 Hz, we then selected the cantilever size.

The fundamental resonant frequency of a cantilever beam is^42^

2 $${f}_{0}=\frac{1}{2\pi }\sqrt{\frac{k}{{m}_{eff}}}$$

where *k* is the cantilever stiffness, and *m*_*eff*_ is the effective mass of the beam.

The addition of a proof mass *M* can be taken into account using the equation^42^:

3 $${f}_{0}=\frac{1}{2\pi }\sqrt{\frac{k}{{m}_{eff}+M}}\mathop{\cong }\limits_{{m}_{eff} <  < M}\frac{1}{2\pi }\sqrt{\frac{k}{M}}$$

The stiffness of the triangular cantilever (V-shaped) can be calculated using the parallel beam approximation (PBA) according to the formula from Butt *et al*.^43,44^; that is,

4 $${k}_{{\rm{\Lambda }}}=\frac{Ed{t}^{3}}{2{h}^{3}}$$

where *E* is Young’s Modulus, *h* represents the triangular beam height, $d=w/\cos \,(\theta /2)$ is the skewed arms width, and θ is the angle between the arms, as shown in Fig. 2(c). The underlying assumption of this approximate formula is that the two beams, forming a triangle with height *h*, can be idealized as a single beam of width 2*d* and length *h*.

By selecting a ferrule borosilicate glass with a transverse section of 3 × 3 mm^2^ and by providing a ridge of 1 × 3 mm^2^ to support the cantilevers (see Fig. 2(c)), the height *h* of the triangular beam is 2 mm, and the two beams can be approximately skewed by an angle θ = π/4.

According to equations (3–4) and considering two borosilicate glass rectangular cantilevers (E = 63 GPa, and ρ = 2230 kg/m^3^) with thickness t = 20 µm and width w = 200 µm (d ≈ 216.5 µm), a proof mass of 17.264 mg is found to be sufficient to set the resonant frequency at 100 Hz (the stiffness is 6.82 N/m).

### Numerical analysis

Of course, the formulas used here rely on a certain degree of approximation, even if they enable a fast design of the mechanical structures^44^. We confirmed the accuracy of the design using finite element method analysis (see the Supplementary Information for details on the numerical analysis)^45^. The structural design obtained using the geometrical parameters previously described is shown in Fig. 2(d). The cantilever elastic properties have been retrieved from the nominal properties of the borosilicate glass (E = 63 GPa, Poisson ratio ν = 0.2, and ρ = 2230 kg/m^3^).

A loss factor of 0.1 has been arbitrarily selected to model the damping loss, as we necessarily postpone the validation of this choice until the experimental stage. Similarly, for simplicity, we numerically modelled the proof mass as a steel sphere (E = 200 GPa, Poisson ratio ν = 0.33, and ρ = 7850 kg/m^3^) with a radius of 806.7 µm to idealize the proof mass of 17.264 mg. However, another proof mass with the same weight could be equivalently adopted.

As an example of the cantilever behaviour, we present in Fig. 2(e) the deformed shape representation of the structure at 5 Hz when subjected to a vertical acceleration of 1 m/s^2^. The ground acceleration (along the z-direction) deflects the cantilever, and the displacement of its free end contains information on the ground acceleration. To retrieve the sensor dynamic response, the displacement is evaluated at the sphere contact point under the cantilever free end. The resulting sensor amplitude and phase responsivity versus frequency is reported in Fig. 2(f,g), respectively. A slight inconsistency appears in the natural frequency, which is 96 Hz instead of 100 Hz. We corrected this disagreement by varying the proof mass size. In the same graph, we display the responsivity obtained numerically with a proof mass of ≈15.91 mg (simulated with a steel sphere with a radius of 785 µm), leading to a natural frequency well matched with the design frequency (i.e., 100 Hz) and an in-band responsivity S_0_ of ≈2.5 µm/(m/s^2^).

### Optimized design

**Figure 3:**
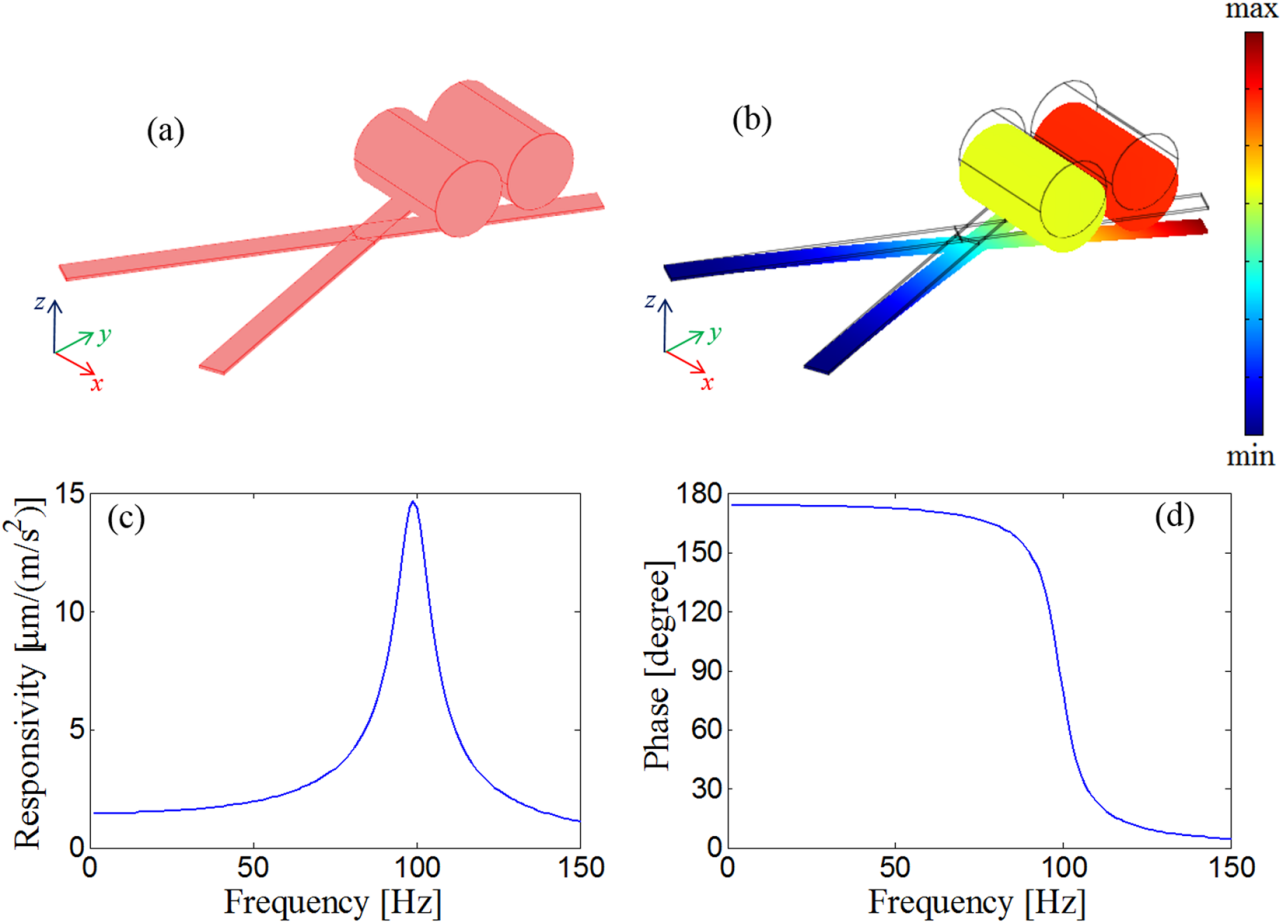
(**a**) Geometry/computational domain of the X-shaped cantilever; (**b**) “deformed shape” representation of the total displacement when the structure is subject to a vertical acceleration at 5 Hz; (**c**) amplitude of the numerical responsivity of the OF sensor; and (**d**) phase of the numerical responsivity of the OF sensor.

As noted, our analysis leaves open two issues that are addressed empirically. The first is the application of a damper layer to increase the overall system damping factor. The second is the appropriate definition of the real proof mass. In place of the steel sphere, we used pieces of copper wire. Copper wires are easily available and can be cut to obtain the desired weight. To obtain a proof mass of 15.91 mg, copper wires with a total length of approximately 6.5 mm should be located at the free end of the V-shaped cantilever. However, to assure a more stable support for the proof mass, we changed the designed structure slightly, replacing the V-shaped cantilever with an X-shaped structure, as shown in Fig. 3(a).

The basic assumptions of the PBA remain valid; thus, equation (4) can be used to estimate the X-shaped beam stiffness provided that the height *h* represents the proof mass contact point and not the cantilever crossing point. By shifting the proof mass 1 mm forward, the copper wires can be easily placed on the bifurcating cantilever arms of the X-shaped beam structure. Furthermore, the mass itself can be reduced to compensate for the decreased stiffness and obtain the same natural frequency.

For the X-shaped cantilever, the proof mass is estimated as follows:

5 $${M}_{X}={M}_{{\rm{\Lambda }}}{(\frac{{h}_{X}}{h})}^{3}$$

If ${M}_{{\rm{\Lambda }}}\cong 15.91\,{\rm{mg}}$, *h*_*X*_ = 3 *mm* and *h* = 2 *mm*, then we have ${M}_{X}\cong 4.71\,{\rm{mg}}$, corresponding to a copper wire length of approximately 1.9 mm.

In Fig. 3(b,d), we display the results of the numerical analysis for the X-shaped cantilevers with two pieces of the copper wire for a total length of 1.916 mm closely positioned 3 mm from the clamped cantilever ends. In particular, in Fig. 3(b) we display the “deformed shape” representation of the total displacement when the structure is subject to a vertical acceleration at 5 Hz, while in Fig. 3(c) and (d) respectively, we show the amplitude and phase of the numerical responsivity of the OF sensor. The cantilever beam natural frequency remains substantially unchanged, whereas the in-band responsivity has been reduced (${S}_{0}\approx 1.5\frac{\mu m}{(m/{s}^{2})}$) because the OF is not located at the maximum deflection point but rather at the crossing region of the cantilevers. Nonetheless, this responsivity reduction is acceptable because the target resolution can still be achieved using interrogation units with a resolution better than $15\frac{pm}{\sqrt{Hz}}$.

Overall, the X-shaped cantilever beam behaves as a mass-spring-damper system, as expected. The simulated structure, featured by the responsivity in Fig. 3(c),(d), represents the designed opto-mechanical cavity to be fabricated on the OF tip. Note that the designed LOF sensor is sensitive exclusively along the z-direction (i.e., it is a uniaxial sensor); accelerations in the transversal plane are neglected. Specifically, the cross-sensitivity, expressed as a percentage of axial sensitivity, is relatively low (10^−4^%) along the x-direction and lower than 0.2% along the y-direction (see the Supplementary Information).

### Fabrication process

**Figure 4:**
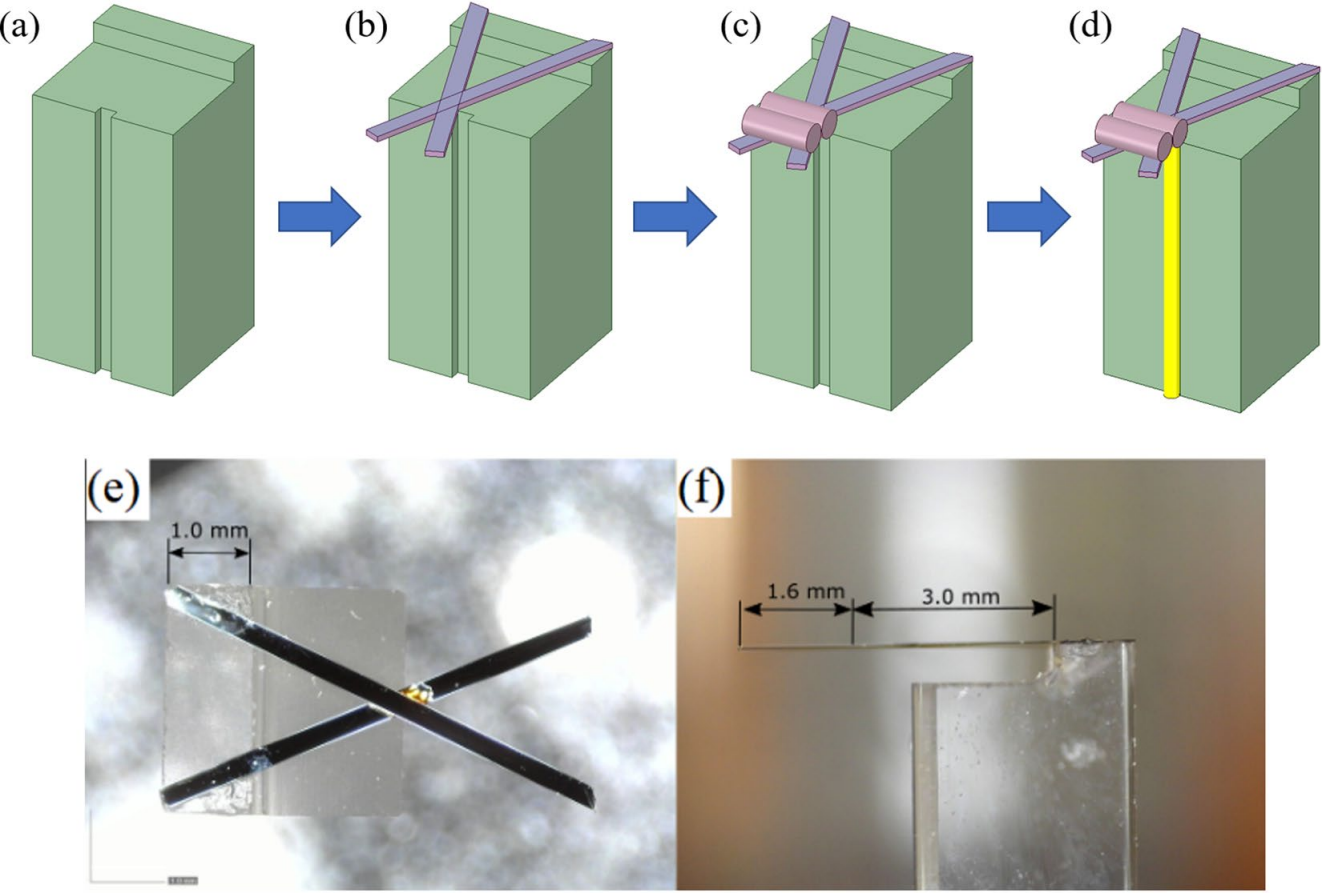
Illustration of the production steps: (**a**) Borosilicate glass ferrule with fabricated ridge and groove; (**b**) cantilevers attached to the ferrule; (**c**) proof mass fixed at the end of the cantilever; and (**d**) readout fibre attached to the sensor. (**e**) Front view of the accelerometer; (**f**) side view of the accelerometer.

The fabrication approach used for the designed LOF seismic sensors was adapted from the field of ferrule-top technology. Early demonstrations of this fabrication route^26,27,29^ involved the carving of a glass ferrule using a ps-laser ablation tool to obtain cantilever-based structures. Here, we adopt the same approach using more flexible and rapid fabrication steps, as shown schematically in Fig. 4(a–d).

The backbone of the structure consists of a 3 × 3 × 7 mm^3^ borosilicate glass ferule. To provide proper support for the sensor a single side ridge of dimensions 1 × 3 mm^2^ is cut out using a customized wire cutter (Well, Model 4240). Successively, one side of the ferrule is modified by grinding a central 200 × 200 µm groove along the longer edge, as shown in Fig. 4(a). This groove provides a guidance for the readout fibre that can be accurately positioned in front the cantilever. Both arms creating the cantilever are made of glass ribbons^46^ with a width of approximately 200 µm and a thickness of approximately 20 µm. The surface of the ribbons is coated by 10 nm and 40 nm layers of chromium and gold, respectively, prior to assembly. The goal of this procedure is to increase the light reflectivity and optimize the quality of the interferometric signal. The beams are fixed to the ferrule using a cyano-acrylate (CA) adhesive, as shown in Fig. 4(b). At the position where the beams cross each other, a small amount of high temperature epoxy (EPO-TEK 353ND) is applied. The epoxy creates a stiff and reliable connection that provides support for the proof mass to be attached in the next step. The top view and side view of one of the fabricated X-shaped cantilevers are shown in Fig. 4(e,f). Figure 4(e) indicates the correct positioning of the cantilevers. The total length of each arm, measured from the base to the tip, is approximately 5.5 mm, and the angle between the arms of the X-shaped cantilever is approximately 45°. In Fig. 4(f), the 3 mm distance from the clamping point, where the proof mass (not shown in the figure) must be applied, is highlighted.

The proof mass, which must be added to tune the sensor response, is composed of two small copper pieces (having a diameter of approximately 0.6 mm and a total length of approximately 1.9 mm) closely positioned at 3 mm, as shown in Fig. 4(c). Additionally, to increase the damping ratio of the structure, a damping layer is applied. The layer, which can also be used to tune the mechanical response, consists of two 20 µm fibres placed along the cantilever beams. The fibres are fixed by a small amount of evenly spread energy dissipative compound (namely, tectyl wax). During fabrication, the resonance frequency of the accelerometer is continuously measured with an external piezo transducer. In the last step, illustrated in Fig. 4(d), an SMF28 single-mode OF is aligned and fixed in the ferrule groove using CA adhesive.

### Data availability

The datasets generated during and/or analysed during the current study are available from the corresponding author on reasonable request.

## Results and Discussion

The fabricated LOF sensors were preliminarily characterized in the laboratory and then integrated into a conventional sensor network for a field trial at the INGV. In the following section, representative data from both laboratory and on-field characterization are reported (while additional details can be found in the Supplementary Information).

### Laboratory characterization

The functionality of the LOF sensors was characterized on an optical table. Specifically, the dynamic response of the LOF sensor was retrieved and compared to an integrated circuit piezoelectric (ICP) accelerometer in the presence of an impulse vibration induced by a hammer blow (see the Supplementary Information for the schematic setup). The responsivity, expressed in terms of amplitude and phase, has been calculated as the ratio between the spectra of the optical sensor output and the reference accelerometer.

The ICP accelerometer was a lead zirconate titanate (PZT) triaxial device (Model 356B18, manufactured by PCB Piezotronics Group Inc., New York, USA) with a (±10%) frequency range of 0.3–5 kHz^37^, whose output was acquired by a dynamic signal analyser (Model RT Pro Photon, BRÜEL & KJÆR, Nærum, Denmark).

The optical sensor displacement was continuously acquired by a commercial interferometric interrogator (Model OP1550 V3, Optics11, Amsterdam, The Netherlands)^40^ to provide the net displacement of the cantilever with a nominal resolution of $3\frac{pm}{\sqrt{Hz}}$^40^. Basically, the interrogator system implements digitally a demodulation technique based on phase-generated carrier method to recover, from the signal reflected by the sensor, the phase shift of the interferometer and thus the desired displacement^47,48^ (see the Supplementary Information for details on the interrogation system).

**Figure 5:**
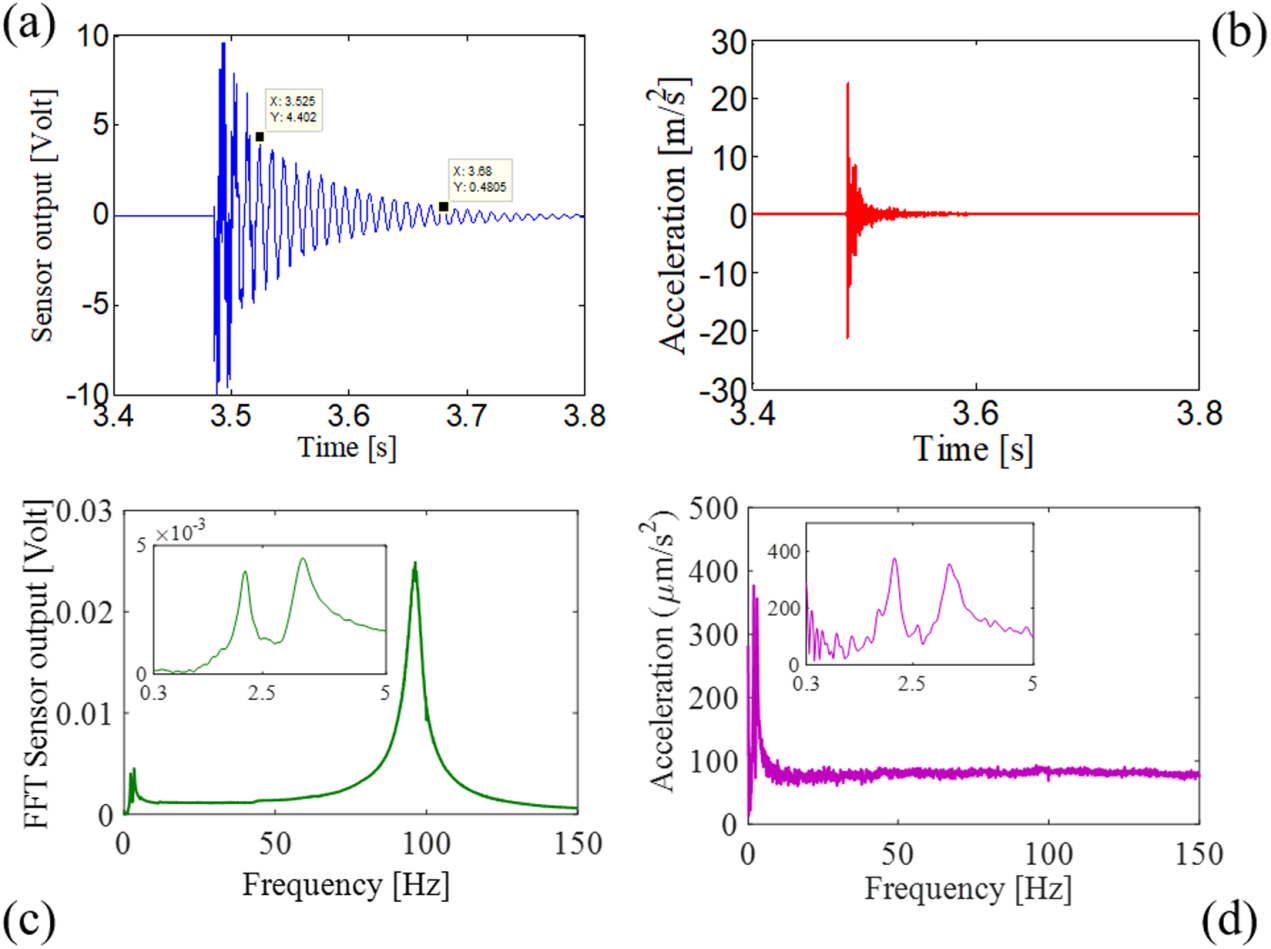
(**a**) Time response of the optical sensor and (**b**) of the PZT sensor to a hammer blow; (**c**) FFT amplitude of the optical sensor response and (**d**) of the PZT sensor response. Inset: zoomed view of the FFT spectra at low frequencies.

Figure 5(a,b) shows the typical response of the optical sensor and reference accelerometer to the hammer blow. The optical sensor response features oscillations at the resonance frequency that exponentially decay. We cannot observe any contribution from the resonance frequency of the commercial accelerometer because the resonance (which is nominally ≥20 kHz) is filtered out. Before retrieving the optical sensor responsivity, information can be obtained by analysing the transient response after the hammer strike. Since the logarithm decrement depends only on the damping ratio, we can experimentally estimate the damping ratio $\varsigma$^49^. By calculating the logarithm decrement Δ over fifteen periods (${\rm{\Delta }}=\frac{1}{N}\,\mathrm{ln}(\frac{{y}_{1}}{{y}_{2}})$ with N = 15), by using the y-axis points from the data tips in Fig. 5(a) and by taking into consideration the relation between the logarithm decrement Δ and the damping ratio $\varsigma$ (${\rm{\Delta }}\approx 2\pi \varsigma$^49^), we estimate the damping ratio to be $\varsigma \approx 0.0235$. Additionally, we can retrieve from the damping ratio both the loss factor $\eta \approx 2\cdot \varsigma =0.047$ and the settling time (at 2%) ${T}_{S}\approx \frac{4}{\varsigma {\omega }_{R}}\approx 0.27s$.

In Fig. 5(c,d), the fast Fourier transforms (FFTs) of both time responses are displayed. The vibration impulse due to the hammer blow features a flat spectrum (with an amplitude of about 100 µm/s^2^) over most of the frequency range of interest, namely, 0–150 Hz. The double peak at low frequency, outlined in the inset of Fig. 5(c) and (d) in both the optical sensor and PZT response, can be attributed to a slight oscillation of the optical table allowed by the hydraulic suspensions. In turn, the hammer blow does not excite significant vibrations (i.e. higher than 100 µm/s^2^)) at frequencies below 2 Hz. Therefore, the sensor characterization is limited to the frequency range of 2–150 Hz.

**Figure 6:**
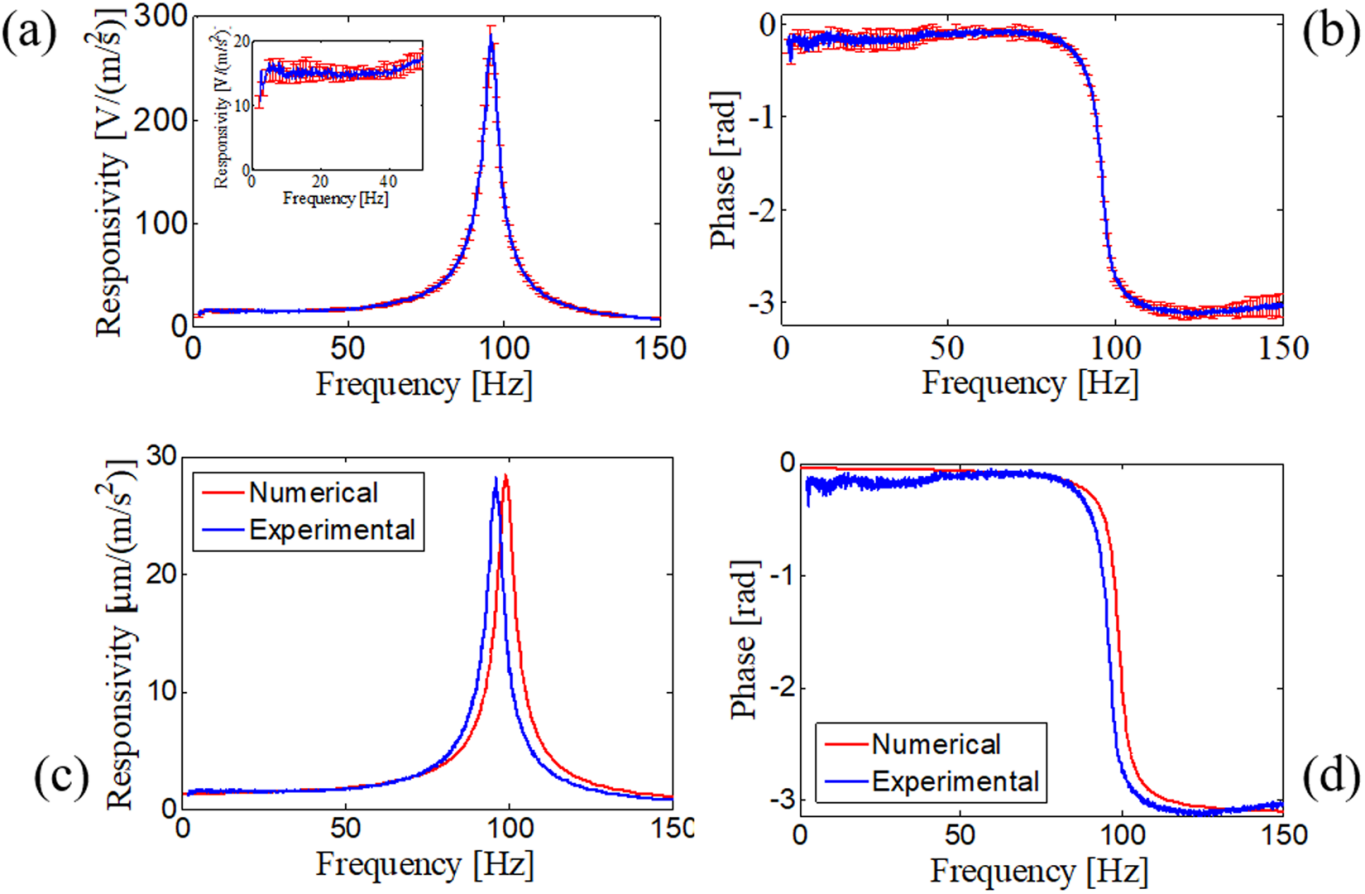
Mean responsivity based on ten measurements and relative error bars for the LOF sensor in terms of the (**a**) amplitude and (**b**) phase. Comparison between the numerical and experimental responsivity of the LOF sensor in terms of the (**c**) amplitude and (**d**) phase.

The amplitude and phase of the sensor responsivity are retrieved as an average of ten successive measurements and are displayed in Fig. 6(a) and (b), respectively. The error bars (in terms of the standard deviation) indicate favourable measurement repeatability.

We now compare the results of the experimental characterization with the numerical predictions. By exploiting the experimental determination of the damping ratio, we can easily incorporate the effect of the damper layer into the numerical simulations. In Figures Fig. 6(c) and (d), we overlap the amplitude and phase of the numerical and experimental responsivity expressed in terms of $\mu m/(m/{s}^{2})$. Except for a small shift of 3 Hz, the experimental characteristic is consistent with the numerical prediction. The shift can be attributed to the damper layer itself, which affects the mechanical resonance, and to imprecisions in the mass determination and positioning. Nonetheless, the fabrication process is reliable and robust and capable of producing the designed structure with negligible discrepancies with respect to the target numerical sensor response.

The responsivity appears particularly flat from 3 Hz up to 40 Hz, as shown in the inset of Fig. 6(a). By taking as the in-band responsivity the mean value of 15 $V/(m/{s}^{2})$ or, equivalently, 1.5 $\mu m/(m/{s}^{2})$, we obtain a 3 dB bandwidth of approximately 60 Hz. In turn, the 10% bandwidth cannot be reasonably measured because of the relative error of approximately 10% in the measurements. Since in the frequency range 0–60 Hz the noise floor level, evaluated as the standard deviation in the time domain (in an urban environment), is typically below 500 µV, we obtain a resolution for detecting seismic waves of 0.44 $\mu g/\sqrt{Hz}$, which is comparable to other commercial seismic accelerometers but with meaningful advantages in terms of size and weight.

### Field trial validation

To demonstrate the capability of the sensor to operate in a seismic network, the sensing system was continuously used by recording the ground acceleration at the INGV. The field trial was exploited to extend the characterization stage at frequencies below 2 Hz, which is a spectral range of utmost importance for seismic wave detection. Specifically, we used the “ground as a shaking table” method^50,51^ to characterize the sensor response at low frequencies. The rationale of this characterization method is essentially the same as the previous one, with the difference that the excitation signals are natural ground vibrations.

To characterize the optical sensor response and to validate the long-time usage of the sensor itself, we placed both the optical sensor and the reference sensor on the ground. The reference sensor is a Kinemetrics Episensor FBA-EST, a state-of-the-art component of seismic stations commonly implemented by INGV for seismic surveillance activities (see the Supplementary Information).

**Figure 7:**
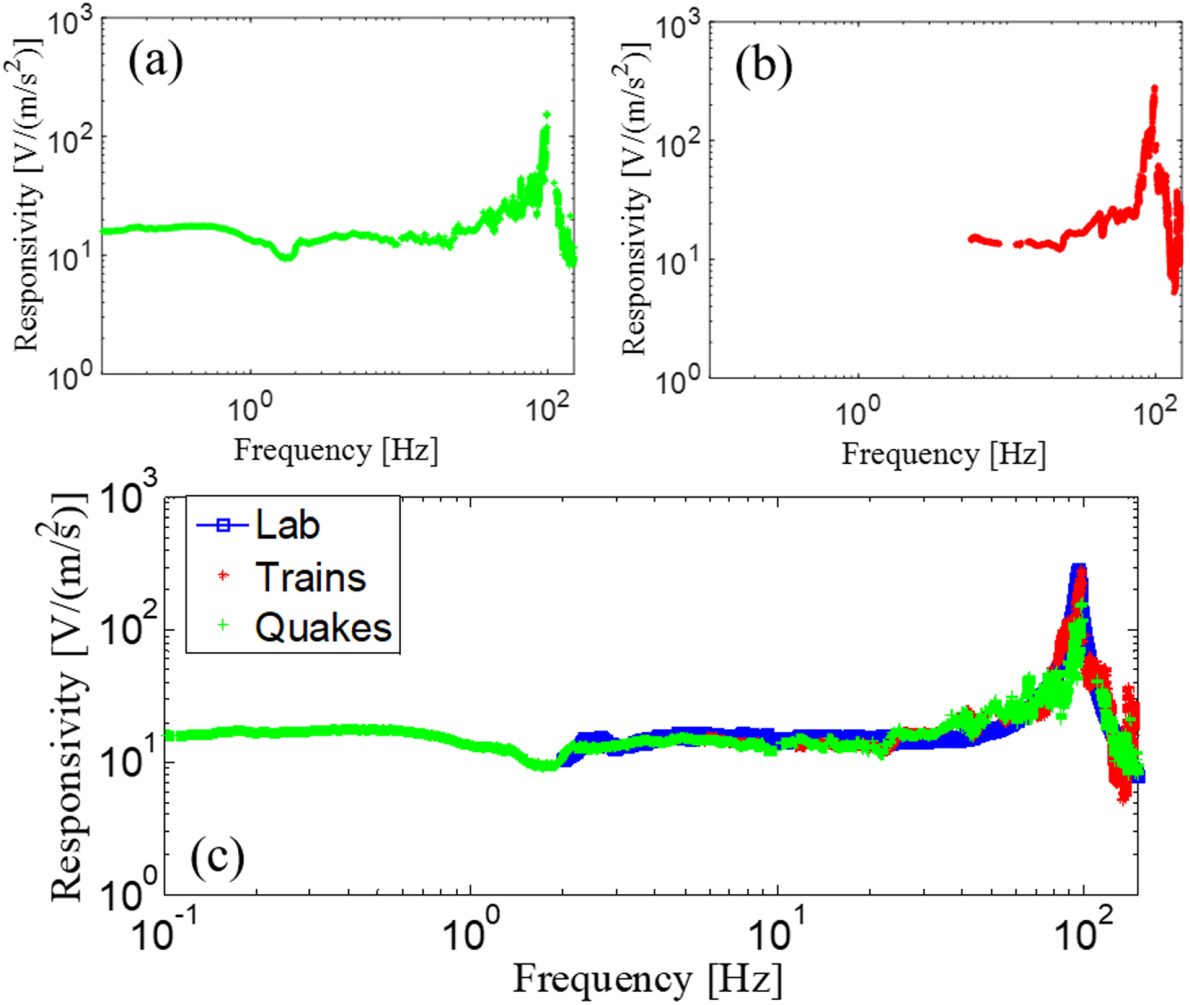
(**a**) Experimental responsivity of the optical fibre sensor retrieved using five earthquakes via the ground using the shaking table method; (**b**) experimental responsivity of the optical fibre sensor retrieved using ten train transits via the ground using the shaking table method. (**c**) Comparison among the experimental responsivities of the LOF sensor.

To compare the spectral features of the optical sensor against the reference, we used traces recorded from five seismic earthquakes that occurred during the acquisition period. These earthquakes are associated with the Amatrice-Norcia seismic sequence^52^. Additionally, as further confirmation, we also selected 10 seismic transients due to local train transits. By using both data sets, we retrieved the lab-on-fiber seismic sensor responsivity (as explained in details in the Supplementary Information), displayed in Fig. 7(a) and (b) respectively, to enable comparison with the laboratory characterization.

The overall results of this second characterization stage are reported in Fig. 7(e). Both characterizations are consistent in the overlapped frequency range and are successful in reducing the lower value of the spectral range of the characteristic response to 0.1 Hz.

### The Norcia earthquake

**Figure 8:**
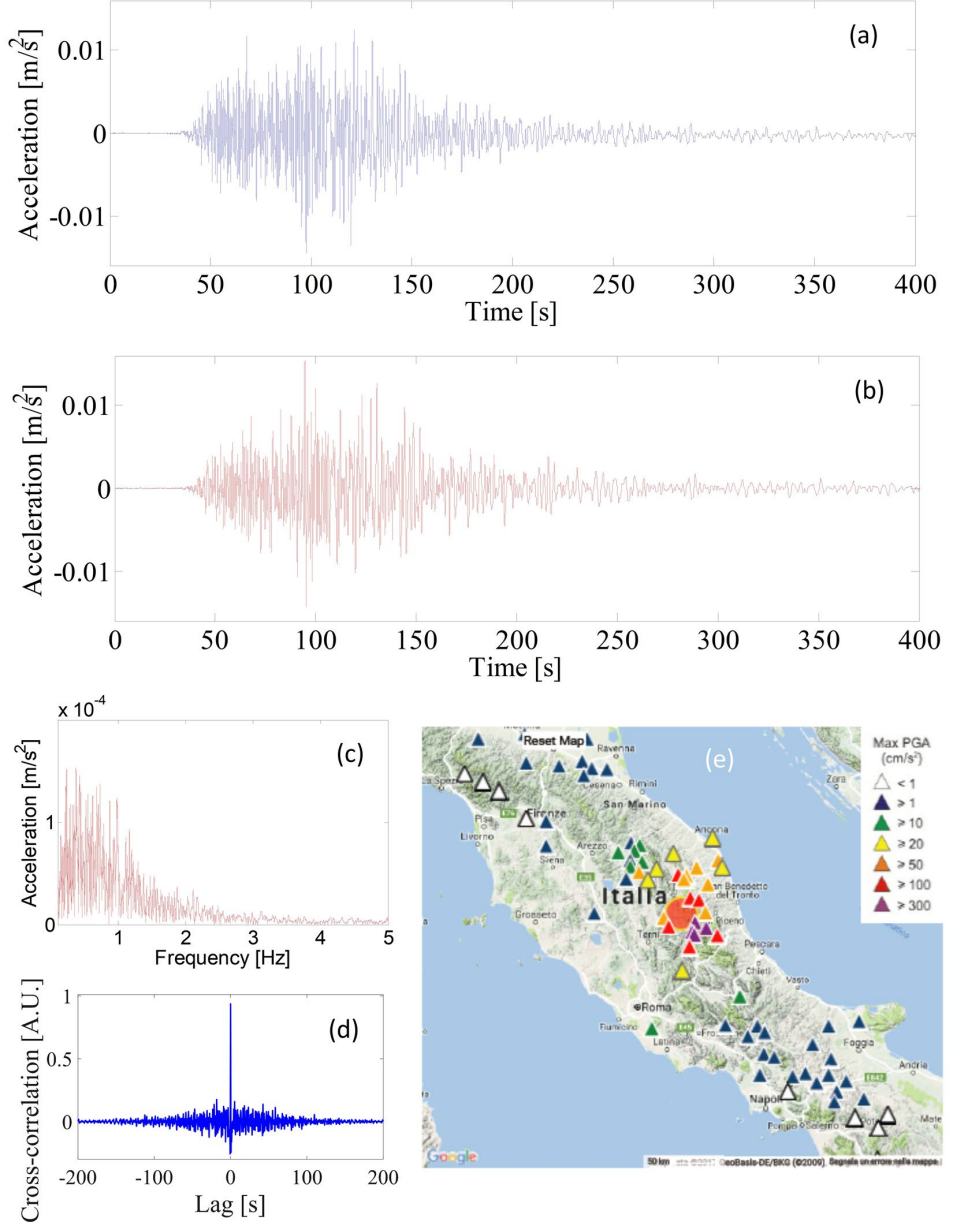
(**a**) Accelerograms of the Mw6.5 Norcia earthquake recorded on the third floor of the INGV-Osservatorio Vesuviano by Episensor FBA-EST Kinemetrics (blue, top panel) and (**b**) by the optical sensor (red, bottom panel); (**c**) FFT spectrum of the Norcia earthquake sensed by the LOF sensor (up to 5 Hz) (**d**) Cross-correlation between the two signals, filtered in the frequency range of 0.1–5 Hz, recorded with the optical sensor and the Episensor (**e**) PGAs recorded by the INGV accelerometric stations during the 30 October (06:40:17), Mw6.5, mainshock (from ISMD 2.0: http://ismd.mi.ingv.it/^54^).

During the field trial, a destructive earthquake struck Norcia, in Italy, on October 30, 2016. The LOF seismic accelerometer (positioned in Naples, Italy) clearly recorded the shock. To show the capability of the optical sensing system to operate as a seismic accelerometer, we compare the accelerograms of the Mw6.5 Norcia earthquake from the Episensor and the optical sensor. In Fig. 8(a) and (b), the vertical components of the Mw6.5 are filtered in the band of 0.1 and 5 Hz for the two sensors. To convert the OF sensor output in terms of acceleration, we use the mean in-band responsivity. Bandpass filtering in the frequency range of 0.1–5 Hz is applied to suppress the noise for both systems because the spectral content of this earthquake is concentrated predominantly over this frequency range (see Fig. 8(c)). In Fig. 8(d), we display the cross-correlation between the two signals recorded with the optical sensor and the Episensor, to show quantitatively the favourable similarity between the sensors.

The measured ground acceleration is consistent with the maximum amplitude values recorded by the INGV seismic network. Ground shaking produced by the Mw6.5 earthquake shows peak ground acceleration (PGA) values of more than 300 cm/s^2^ recorded by the 4 stations close to the epicentre and peak acceleration values of less than 1 cm/s^2^ recorded by the stations approximately 300 km away (see Fig. 8(c)).

**Figure 9:**
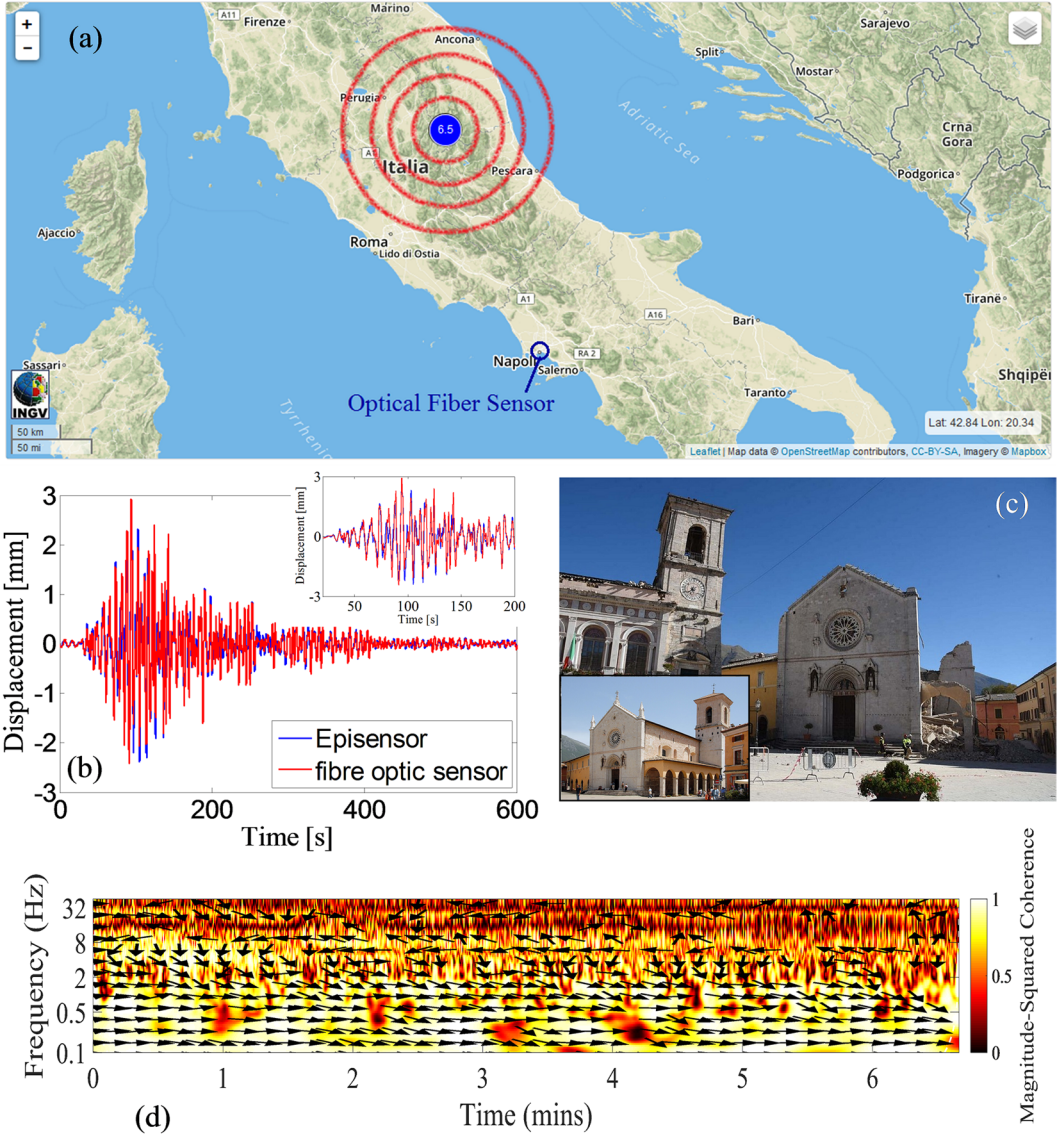
(**a**) Map of the Norcia earthquake epicentre retrieved from the INGV official website (http://cnt.rm.ingv.it/, the map is released under a CC-BY-SA licence https://creativecommons.org/licenses/by-sa/3.0/); (**b**) comparison between the ground displacement traces of the LOF sensors and the reference sensor; (**c**) photograph of St. Benedict Cathedral in Norcia after the earthquake^55^; in the inset, a picture of the Cathedral before the earthquake is displayed (the picture is released under a CC-BY-SA licence https://creativecommons.org/licenses/by-sa/3.0/ by Wikimedia^56^); (**d**) wavelet coherence between the optical and Episensor displacement signal over a 400 s window.

In Fig. 9, we show the ground displacement retrieved by double integration of the accelerograms over the full sensor bandwidth of 0.1–60 Hz. The two traces overlap in Fig. 9(b), highlighting their similarity either in duration or in amplitude. The magnified view of the ground displacement in the inset of Fig. 9(b) allows validation and checking of the polarity of both sensors by comparing the shape of the first pulses. From the displacement traces, the duration of the Norcia earthquake (approximately six minutes, well over the limit of human perception) can be better appreciated. We emphasize that the LOF sensor was located several kilometres from the epicentre, as shown by the map in Fig. 9(a).

Finally, a quantitative evaluation of the similarity between the two signals was conducted using the coherence in the time and frequency domains^53^ for the temporal window (0–400 s) displayed in Fig. 9(b). Figure 9(d) shows the wavelet coherence between the two signals transformed in displacement from acceleration^53^. The colour scale corresponds to the normalized coherence magnitude, whereas the arrow directions indicate the phase lag between the signals on the unit circle. When the arrows are directed towards the right, the signals are essentially in phase. As evidenced by colour contouring, the coherence between signals is high for frequency values up to 2 Hz; within this frequency range, the ground displacement is significantly larger than the noise floor. As indicated by the arrows, the phase lag over the same frequency range is negligible, demonstrating the high fidelity of the LOF sensors for seismic wave detection.

## Conclusions

A novel LOF seismic accelerometer has been successfully demonstrated. The sensor is composed of a micro-opto-mechanical cavity on the fibre tip, forming an extrinsic Fabry-Pérot cavity with the fibre end facet. The micromechanical structure, featuring a dual-beam cantilever with a proof mass, was designed to exhibit competitive performance for seismic applications. Prototype sensors were fabricated using ferrule-top technology, and a commercial interferometric interrogation system was used to record the displacement of the dual-beam cantilever. The responsivity was preliminarily measured in the laboratory, demonstrating a resolution down to 0.44 $\mu g/\sqrt{Hz}$ over a 3 dB frequency band of 60 Hz. To demonstrate the sensor capability, the sensing system was continuously used in combination with a commercial seismic sensor for two weeks. By comparing the ground acceleration sensed by the OF seismic sensor and using a commercial seismic accelerometer, the sensor responsivity was retrieved, extending the sensor characterization at low frequencies down to 0.1 Hz. During the trial, the OF sensor accurately sensed and registered the ground acceleration associated with the earthquakes that occurred near the end of October and early November 2016 in Central Italy. The wave traces were compared with the recordings of a traditional sensor. The time correlation and spatial coherence are used to show the high fidelity of the LOF sensors in seismic wave detection.

Additionally, the OFT offers a wide variety of benefits in deploying such a sensor in a real seismic network. The lightness and small size of the LOF sensors and the immunity of the OF towards electromagnetic interferences enable a simpler installation than that of traditional sensors. The OF sensor performance and the effectiveness in detecting ground acceleration during earthquakes, combined with the benefits of OFT, establish the proposed seismic accelerometer as a promising and valuable alternative to standard seismic accelerometers.

## Electronic supplementary material

Supplementary Material
