# Supplementary material for: Opto-mechanical lab-on-fibre seismic sensors detected the Norcia earthquake
Source: Sci Rep. 2018 Apr 27;8:6680. doi: 10.1038/s41598-018-25082-8 (PMC5923338; doi:10.1038/s41598-018-25082-8)
Supplement: Supplementary file 1 — Supplementary Material [file 41598_2018_25082_MOESM1_ESM.pdf]

# Supplementary Material

# Opto-mechanical lab-on-fibre seismic sensors detected the Norcia earthquake

*Marco Pisco* [a], *Francesco Antonio Bruno* [a], *Danilo Galluzzo* [b], *Lucia Nardone* [b], *Grzegorz Gruca* [c], *Niek Rijnveld* [c], *Francesca Bianco* [b], *Antonello Cutolo* [a], *Andrea Cusano* [a,*]

[a] Optoelectronic Division - Engineering Dept., University of Sannio, c.so Garibaldi 107, 82100 Benevento, Italy.

[b] Istituto Nazionale di Geofisica e Vulcanologia, Osservatorio Vesuviano, via Diocleziano 328, 80124, Napoli.

[c] OPTICS11 B.V., De Boelelaan 1081, 1081 HV Amsterdam, The Netherlands.

**Abstract:** This document contains additional details on the design, fabrication and experimental characterization, as well as supplementary results. Newly introduced equations are labeled with the prefix “S”, whereas newly introduced figures and tables are referred to as “Supplementary” and are labeled with the prefix “S”; all others pertain to the main text. All references are intended as local, and those already utilized in the main text are repeated.

## Supplementary information on the fibre optic seismic sensor modelling

A flexible cantilevered beam with a mass affixed on the end, schematically sketched in Figure S1(a), can be modelled as a mass-spring-damper system, as shown in Figure S1(b). The relation between the acceleration with respect to the ground and the cavity length variation (Figure S1(c)) can be retrieved analytically. Starting from Newton's law of motion, a mass-spring-damper system is modelled according to the following equation [S1]:

$$\frac{\partial^2 x(t)}{\partial t^2} + 2\varsigma\omega_R \frac{\partial x(t)}{\partial t} + \omega_R^2 x(t) = \frac{\partial^2 u(t)}{\partial t^2} = a(t) \qquad \text{(S1)}$$

where

*x(t)* is the mass displacement with respect to the ground versus time *t* (defined as positive for a downward mass motion),

*u(t)* is the ground displacement (defined as positive for an upward ground motion),

*a(t)* is the ground acceleration to be detected,

$\omega_R = \sqrt{\frac{k}{m}}$ is defined as the natural frequency and $\varsigma = \frac{c}{2\sqrt{k \cdot m}}$ as the damping ratio,

*m* is the mass, *k* the spring constant, and *c* is the viscous damping.

The system dynamic response can be obtained by transforming equation (S1) in the Fourier domain and by retrieving the ratio between the mass displacement *X(ω)* and the ground acceleration to be detected *A(ω)* with respect to the ground versus harmonic frequency ω:

$$H(\omega) = \frac{X(\omega)}{A(\omega)} = \frac{1/\omega_R^2}{1 - \left(\frac{\omega}{\omega_R}\right)^2 + \left(2\varsigma\frac{\omega}{\omega_R}\right)j} \quad \text{(S2)}$$

Figure S1(d-e) shows the harmonic response ($H(\omega)$), in amplitude and phase, normalized to the responsivity $S_0 = 1/\omega_R^2$ versus the harmonic frequency $\omega$ normalized to the natural frequency $\omega_R$ for various damping ratio values.

Figure S1(f) shows the cut-off frequencies obtained by imposing the squared normalized amplitude equal to the squared ($S_0$ ± 10% $S_0$) and ($S_0$ ± 50% $S_0$). The minimum cut-off frequency represents the normalized flat bandwidth at 10% and 50% (3 dB bandwidth) for various damping ratio values. For a slightly damped system (i.e., $\varsigma \leq 0.1$), the bandwidth does not depend on the damping ratio.

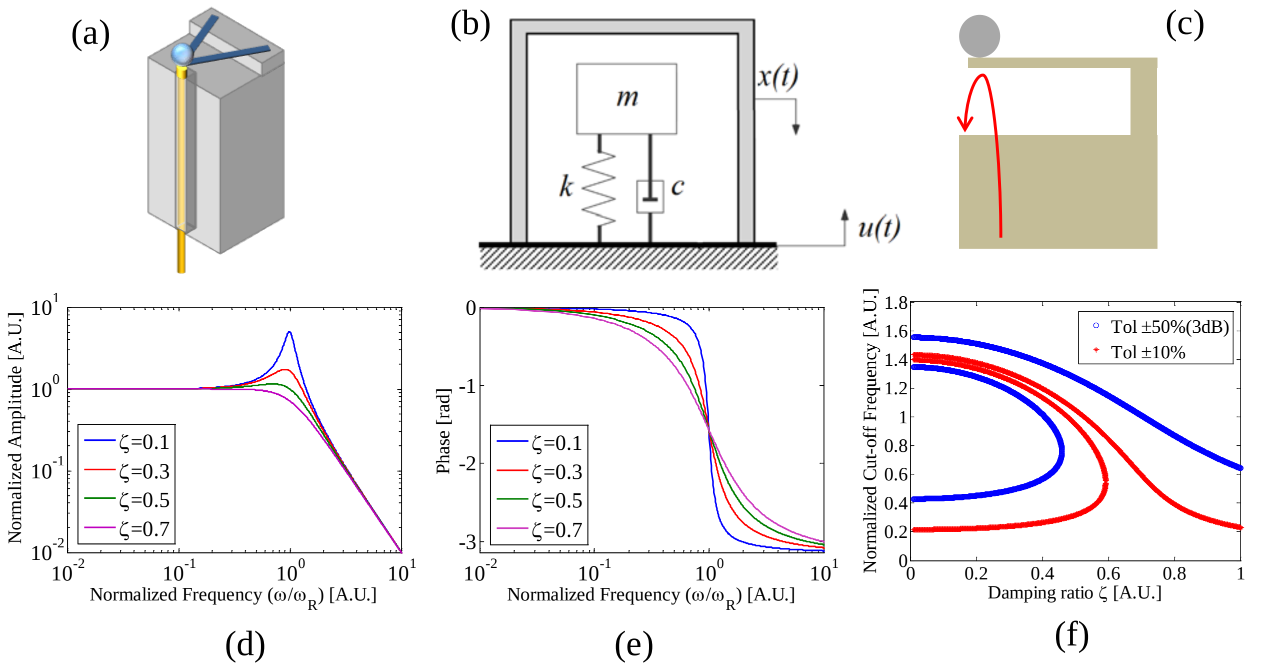


**Figure S1** *(a) Schematic of the fibre optic seismic sensor; (b) diagram of a generic mass-spring-damper system; (c) schematic (side view) of the interferometric cavity formed by the optical fibre cleaved end and the cantilever free end; (d) normalized amplitude and (e) phase of the system dynamic response versus the normalized harmonic frequency for various values of the damping ratio; and (f) normalized cut-off frequency calculated with a tolerance of 50% and 10% versus the damping ratio.*

## Supplementary information on the numerical analysis

The mechanical response of the opto-mechanical cavities discussed in the main paper was analysed using Finite Element Method (FEM) analysis. Specifically, we used the solid stress-strain model in the structural mechanics module in COMSOL Multiphysics® [S2] to numerically estimate the frequency response of the V-shaped cantilever with an added mass at the free end when the cantilever is subjected to an uniaxial acceleration longitudinal to the fibre axis.

After drawing the computational domain (in agreement with the geometrical parameters provided in the main paper), the physical properties of the constituting materials were selected in the subdomain settings. The properties of the materials used in the simulations, namely, borosilicate glass, steel and copper, are included in the default materials library.

The unitary acceleration was set to 1 $m/s^2$ along z using the load menu in Cartesian coordinates.

The computation domain has no constraints with the exception of the cantilever clamped ends, to which a fixed boundary setting was applied. After applying a normal mesh, the simulation was conducted for frequencies up to 150 Hz with 1 Hz steps using the direct (spools) solver.

To retrieve the sensor dynamic response, the displacement was evaluated in the symmetry point (in the point evaluation menu) under the cantilever free end.

## Supplementary information on the optimized design: transverse cross-sensitivity

The transverse cross-sensitivity of the sensor is numerically calculated with the same model previously described. In this case, the unitary acceleration was set to 1 $m/s^2$ along x and y, respectively.

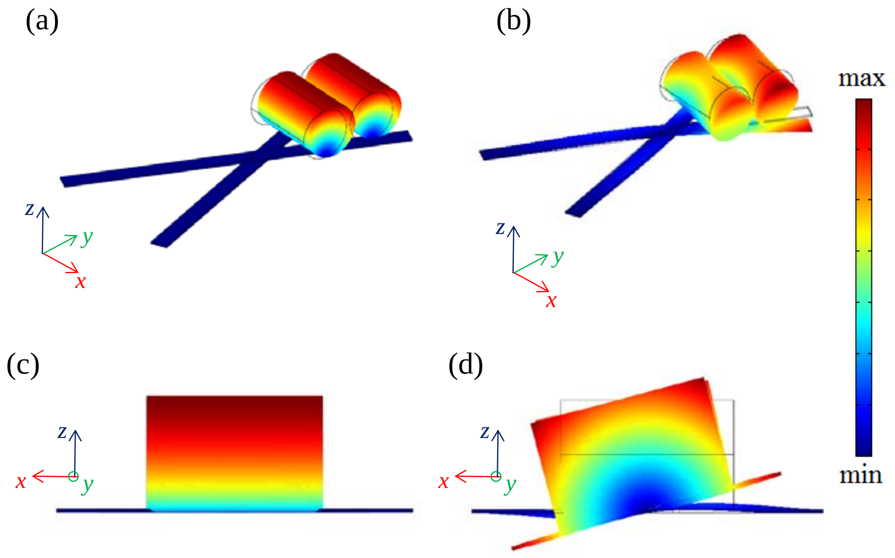


**Figure S2** *"Deformed shape" representation of the total displacement, when the structure is subject to a transverse acceleration at 5 Hz (a) along the y-direction and (b) the x-direction. The same representation is displayed in the xz-plane for the transverse acceleration at 5 Hz (c) along the y-direction and (d) the x-direction.*

In Figure S2, as an example, we display the “Deformed shape” representation of the total displacement, when the structure is subject to a transverse acceleration at 5 Hz along the x and y direction. In Figure S3, the transversal cross-sensitivity, resulting from the numerical analysis of the designed sensor, is displayed and it is expressed as a percentage of axial sensitivity, along the x-direction and the y-direction.

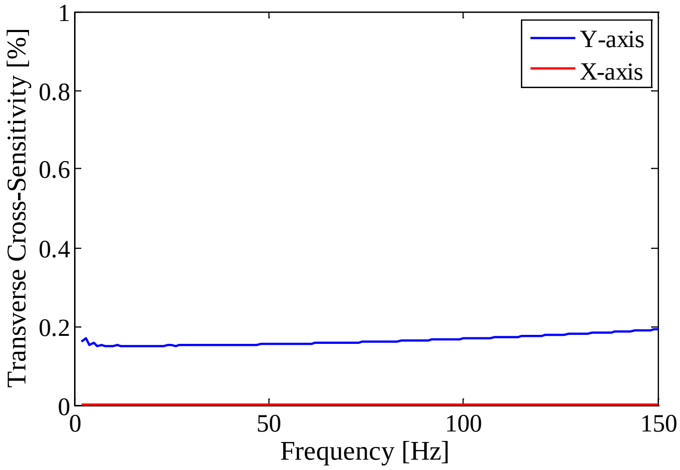


**Figure S3** *Transversal cross-sensitivity, expressed as a percentage of axial sensitivity, along the x-direction and the y-direction*

## Supplementary information on the spectral characterization

As last step of the fabrication process, the fibre positioning is performed by exploiting the groove created in the ferule. After each fabrication, the spectral characterization of the sensor is carried out. In figure S4, the scheme of the setup for the spectral characterizations is displayed. The resulting reflectance spectrum of one of the fabricated lab-on-fibre sensor is shown in figure S5. From the fringe pattern of the sensor reflectance, we can estimate the air cavity length to be approximately 329 µm. The air cavity length indeed can be easily estimated by modelling the interferometer as a Fabry-Pérot and by assuming a refractive index of 1.45 for the optical fibre and the cantilever.

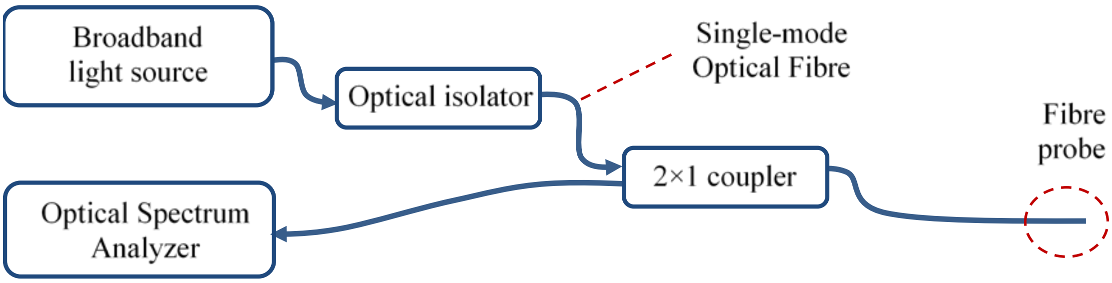


**Figure S4** *Schematic of the experimental configuration used for reflectance spectra characterization.*

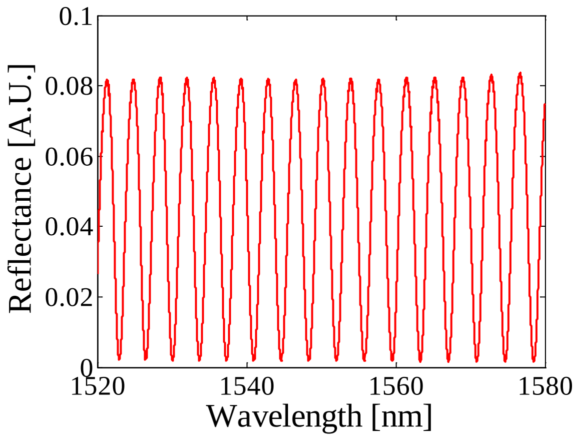


**Figure S5** *Reflectance spectrum of a lab-on-fibre seismic accelerometer.*

## Supplementary information on the laboratory characterization

In figure S6, the experimental setup arranged on an optical table and used for the responsivity characterization is displayed. During measurements, the hydraulic suspensions of the table were maintained in an active state to suppress background vibration.

As reported in the main text, the ICP accelerometer was a lead zirconate titanate (PZT) triaxial device (Model 356B18, manufactured by PCB Piezotronics Group Inc., New York, USA) with a (±10%) frequency range of 0.3-5 kHz [S3]. The optical sensor displacement was continuously acquired by a commercial interferometric interrogator (Model OP1550 V3, Optics11, Amsterdam, The Netherlands) [S4] to provide the net displacement of the cantilever. A conversion factor of 0.1 µm/V was used. The sensor output was represented on a full scale of 20 V (i.e., -10 to 10 V). The interrogation unit also provides an analogic voltage output proportional to the measured displacement.

The electrical output of the two sensors was connected to a dynamic signal analyser (Model RT Pro Photon, BRÜEL & KJÆR, Nærum, Denmark) for synchronous signal digitization and storing.

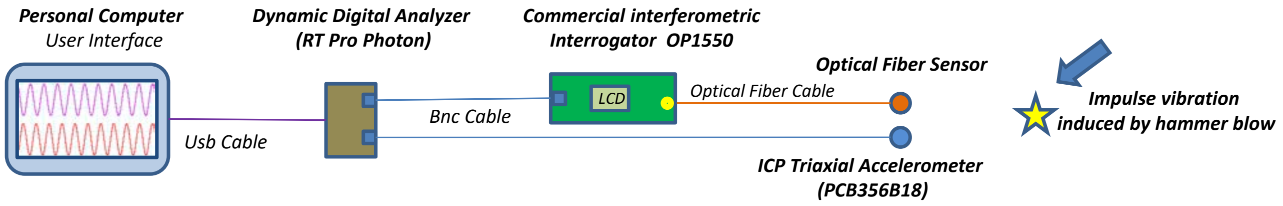


**Figure S6** *Experimental configuration for optical sensor characterization in the laboratory.*

## Supplementary information on the interrogation unit

The interrogation unit is equipped with an integrated tuneable laser (Sampled Grating Distributed Bragg Reflector, SGDBR, laser), operating in the spectral range 1528-1563nm. The source laser wavelength is modulated around 1530nm by injecting in the laser phase section a modulated current of about 2mA at 150 kHz. As a result, the carrier frequency modulation spans over a bandwidth of

about 0.2nm. The modulated signal reflected by the seismic sensor is sent to a photodiode (Hamamatsu G9801-32). A Texas Instruments ADC converter (model ADS8555) digitalizes the output of the photodiode at 800kS by 16bit. A digital signal processing technique is implemented on a Field Programmable Gate Array, FPGA (Cyclone IV - EP4CE15F17C7N), to retrieve the desired phase shift, according to a demodulation scheme based on phase generated carrier approach [S5, S6].

The signal processing involves the detection of the “low” frequency component [S5] and of the signal amplitude at the carrier’s fundamental frequency. Those two orthogonal components are normalized in respect to their maxima and minima. This step is necessary in order to perform precise phase demodulation. The output phase of the interferometer is calculated by the inverse tangent function implemented in the FPGA fabric. The resulting phase is stored in a global 64-bit phase accumulator. Additionally, a fringe counting (i.e. phase unwrapping) is digitally implemented on top of the accumulator – its value is corrected every time when a phase wrap occurs (every $\pi$ radians). This feature allows one to extend the range of demodulation over multiple fringes. In order to provide an analog signal output, the 64-bit accumulator is scaled down to 16 bits based on current sensitivity setting and send to the DAC (DAC8822) at a rate of 800kHz.

**Supplementary information on the field trial validation**

To demonstrate the sensor capability, the sensing system was continuously used in combination with a commercial seismic sensor for two weeks. The sensor responsivity was retrieved by using the ground as a natural shaking table. This method [S7] is applicable to frequency ranges where the seismic signals significantly exceed the sensor noise floor. Furthermore, the two instruments to compare must be exposed to spatially coherent vibration signals. To characterize the optical sensor response and to validate the long-time usage of the sensor itself, we placed both the optical sensor and the reference sensor on the ground, as shown in Figure S7. The reference sensor is a state-of-the-art component of seismic stations commonly implemented by INGV for seismic surveillance activities.

Specifically, we used a standard digital seismic station Reftek 130 equipped with a three-component accelerometer Kinemetrics Episensor FBA-EST. The Episensor used in the experimental configuration has a flat frequency response between DC and 200 Hz and a maximum acceleration equal to 0.25 g. For both sensors (optical and Episensor), the data were recorded with a sample rate equal to 500 Sps in continuous acquisition mode. The instruments were installed on the third floor of the INGV-Osservatorio Vesuviano edifice in the town of Napoli, Italy. In the investigated frequency range, the excitations can be considered spatially coherent, as the sensors were closely

positioned. To detect signals that significantly exceeded the noise floor, the seismic records were acquired with no interruption for 15 days starting on October 24, 2016.

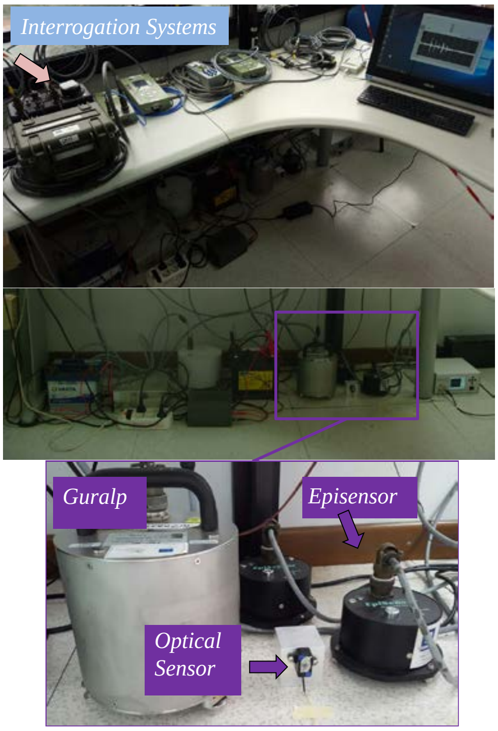


**Figure S7** *Experimental configuration for optical sensor characterization from the ground through the shaking table method*

To compare the spectral features of the optical sensor against the reference, we used traces recorded from five seismic earthquakes that occurred during the acquisition period at a distance of more than 300 km from the installation site (see Table SI). These earthquakes are associated with the Amatrice-Norcia seismic sequence [S8]. Additionally, as further confirmation, we also selected 10 seismic transients due to local train transits.

<table>
<tr><th colspan="7">Earthquakes</th><th></th></tr>
<tr><th rowspan="2">Origin Time (UTC)</th><th rowspan="2">Lat. [deg]</th><th rowspan="2">Long. [deg]</th><th rowspan="2">Depth (km)</th><th colspan="2">Magnitude</th><th rowspan="2">Epicentral Location</th><th rowspan="2">Label</th></tr>
<tr><th>Type</th><th>M</th></tr>
<tr><td>2016-10-26 17:10:36.34</td><td>42.8802</td><td>13.1275</td><td>8.7</td><td>Mw</td><td>5.4</td><td>3 km SW Castelsantangelo sul Nera (MC)</td><td rowspan="2">Quake1</td></tr>
<tr><td>2016-10-26 19:18:05.85</td><td>42.9087</td><td>13.1288</td><td>7.5</td><td>Mw</td><td>5.9</td><td>3 km NW Castelsantangelo sul Nera (MC)</td></tr>
<tr><td>2016-10-28 20:02:43.15</td><td>39.2672</td><td>13.5468</td><td>481</td><td>ML</td><td>5.8</td><td>Tirreno Meridionale</td><td>Quake2</td></tr>
<tr><td>2016-10-30 06:40:17.36</td><td>42.8322</td><td>13.1107</td><td>9.2</td><td>Mw</td><td>6.5</td><td>5 km NE Norcia (PG)</td><td>Quake3</td></tr>
<tr><td>2016-11-01 07:56:40.34</td><td>42.9902</td><td>13.1345</td><td>8.3</td><td>Mw</td><td>4.8</td><td>5 km N Ussita (MC)</td><td>Quake4</td></tr>
<tr><td>2016-11-03 00:35:01.29</td><td>43.0277</td><td>13.0493</td><td>8.1</td><td>Mw</td><td>4.7</td><td>2 km S Pieve Torina (MC)</td><td>Quake5</td></tr>
<tr><td colspan="7">Train Transits</td><td></td></tr>
<tr><td colspan="4">Start Time</td><td colspan="3">End Time</td><td></td></tr>
<tr><td colspan="4">2016 10 25 03:18:07 UTC</td><td colspan="3">2016 10 26 04:50:27 UTC</td><td></td></tr>
</table>

**Table SI.** *Earthquake features and selected time interval of train transits used for the optical sensor characterization.*

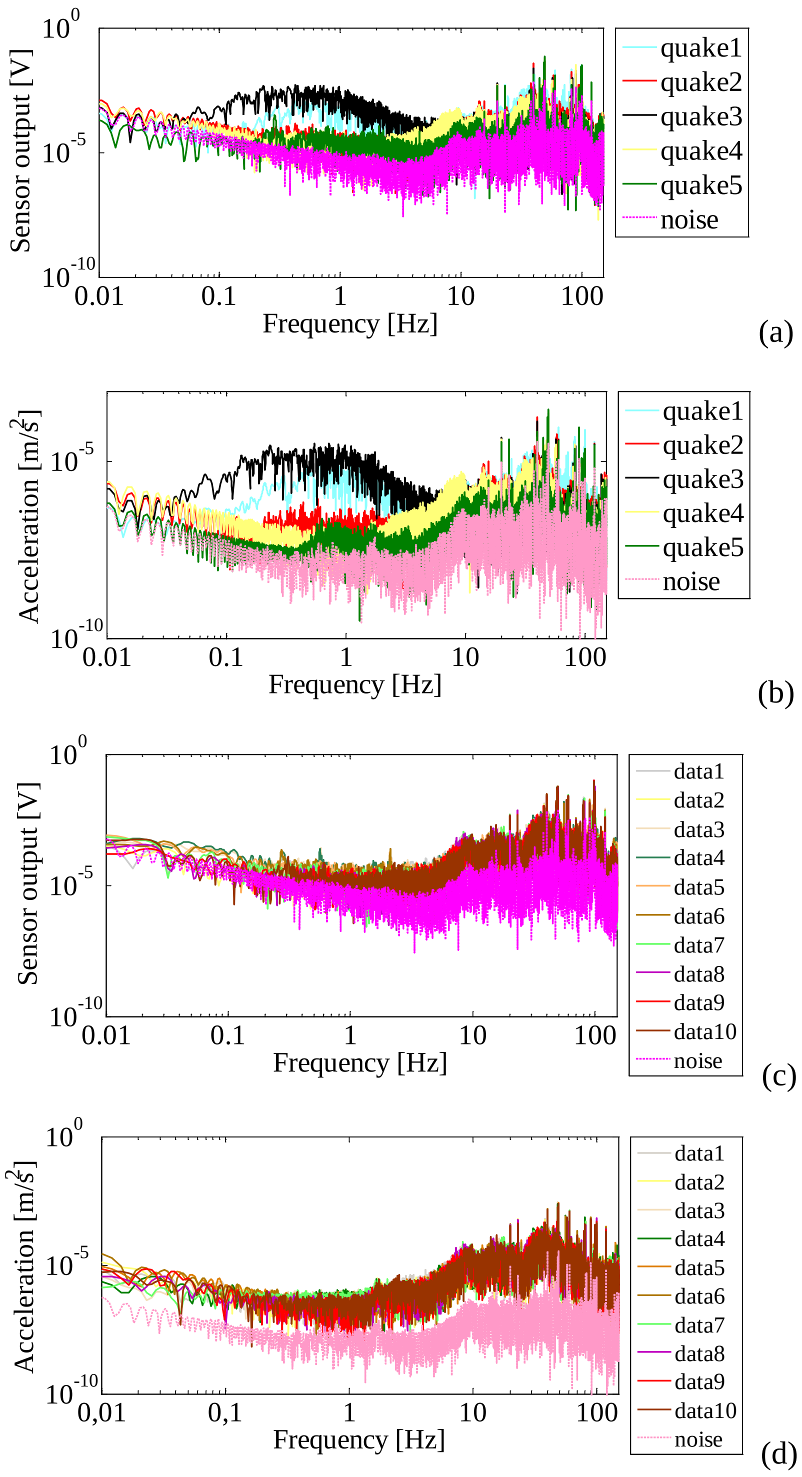


**Figure S8** *(a) Spectra of the optical sensor output and (b) of the reference sensor output for five earthquakes; (c) spectra of the optical sensor output and (d) of the reference sensor output for ten train transits.*

Figure S8 shows the spectra of the five earthquakes and ten train transits over a 3-minute window for the optical sensor and the reference sensor.

By comparing one to one in Figure S8(a) and S8 (b) the earthquakes sensed by the optical sensor and by the reference, clear agreement can be observed. In the same plot, we also show the sensor noise in the presence of low seismic background, registered over the same period during the trial. The noise floor increases at low frequencies by limiting the sensing system performance at low

frequencies. For the combined effect of the optical system noise and of the available seismic vibrations, we set a lower cut-off frequency at 0.1 Hz since the signal-to-noise ratio below this threshold was unsuitable.

For evaluation of the responsivity, we used frequency bands for which the signal-to-noise ratio is more than 10. This approach was necessary to avoid weighting in the responsivity of the noise floor characteristics of the various readout units.

Despite the similarity between the spectra, by calculating the responsivity, the ratio between the two spectra leads to sharp variations. For clarification, we show in Figure S9 (a) a magnified view of the spectra of the sensor output concerning earthquake 1 in the range of 10-20 Hz and the resulting ratio. The ratio varies significantly because of slight shifts in the spectral minima that in turn can be attributed to the different acquisition systems (i.e., a slight difference in the sampling frequency). As confirmed by the previous characterization, such rapid variations in the spectrum are not a feature of the sensors, but they can be attributed to the source ground vibration and thus should be eliminated from the sensor responsivity.

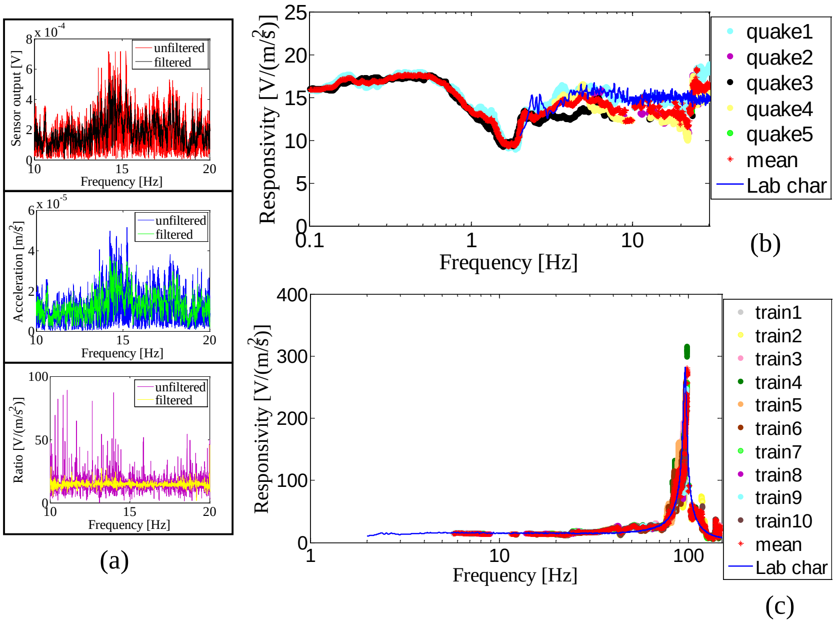


**Figure S9** *(a) Exemplification of the effect of the cepstrum filtering technique to average out the spectra; (b) experimental responsivity of the optical fibre sensor retrieved using five earthquakes via the ground using the shaking table method; (c) experimental responsivity of the optical fibre sensor retrieved using ten train transits via the ground using the shaking table method.*

A solution to a similar problem is offered by sound speech analysis, where the spectral envelope is extracted by the signal to retrieve the voice characteristics independently from the speech (i.e., the source sound). Sharp variations in the source signal are separated by the spectral envelope by means of the cepstrum technique. Similarly, we are interested in the responsivity without care for the sharp spectral variations of the source signal. Thus, before calculating the responsivity, we filtered the

spectra using the cepstrum technique. We selected a normalized frequency of 0.18 as the cut-off frequency to average out the spectral fluctuations without drastically affecting the spectral shape. As an example of the filtering effect, in Figure S9 (a) we show a magnified view of the spectra before and after filtering.

The resulting responsivity of the optical accelerometer evaluated using spectral ratios on earthquakes is shown in Figure S9 (b) to enable comparison with the laboratory characterization.

The characteristic response is not flat as numerically expected because of a dip in the sensitivity at approximately 1.5-2 Hz. We have not ascertained if this dip is a feature of the sensor or of the interrogation unit; in any case, however, the dip affects the optical chain.

We also analysed the train transit spectra displayed in Figures S8 (c) and S8 (d). The train transit spectra typically contain spectral content at frequencies higher than that of earthquakes. Additionally, the spectral curves are strongly overlapping due to the repetition of the same behaviour. The resulting characterization is shown in Figure S9 (c) to enable comparison with the laboratory characterization. Actually, the characterization using the train transit does not add information to the previous responsivity; rather, it is noisy relative to the laboratory characterization results. However, this characterization does serve to confirm previous results and is a demonstration of the functionality of the sensor in a practical scenario.

## References


S1. C. M. Harris and C. E. Crede, Ed., Shock & Vibration Handbook. New York: McGraw-Hill, 1976.

S2. COMSOL Multiphysics, User’s Guide

S3. Datasheet of the accelerometer PCB Model 356B18 - PCB Piezotronics http://www.pcb.com/Products.aspx?m=356B18_ACS-4T (Online on 12 March 2018)

S4. http://optics11.com/products/op1550 (Online on 12 March 2018)

S5. H. van Hoorn, N. A. Kurniawan, G. H. Koenderink, and D. Iannuzzi, “Local dynamic mechanical analysis for heterogeneous soft matter using ferrule-top indentation,” Soft Matter 12, 3066–3073 (2016);

S6. A. Dandridge, A. B. Tveten and T. G. Giallorenzi, "Homodyne Demodulation Scheme for Fiber Optic Sensors Using Phase Generated Carrier," in IEEE Transactions on Microwave Theory and Techniques, vol. 30, no. 10, pp. 1635-1641, Oct. 1982. doi: 10.1109/TMTT.1982.1131302

S7. Havskov, J., Alguacil, G. (2014), “Instrumentation in earthquake seismology”, Springer, 2004.

S8. ReLUIS-INGV Workgroup (2016), “Preliminary study on strong motion data of the 2016 central Italy seismic sequence V6”, available at http://www.reluis.it. (Online on 12 March 2018)
